# Optimising the delivery of breast cancer risk assessment for women aged 30–39 years: A qualitative study of women’s views

**DOI:** 10.1177/17455057231160348

**Published:** 2023-03-31

**Authors:** Sarah Hindmarch, Louise Gorman, Rhiannon E Hawkes, Sacha J Howell, David P French

**Affiliations:** 1 Manchester Centre for Health Psychology, Division of Psychology and Mental Health, School of Health Sciences, Faculty of Biology, Medicine and Health, University of Manchester, Manchester, UK; 2 NIHR Greater Manchester Patient Safety Translational Research Centre and Manchester Academic Health Science Centre, University of Manchester, Manchester, UK; 3 Division of Cancer Sciences, Faculty of Biology, Medicine and Health, Manchester Academic Health Science Centre, University of Manchester, Manchester, UK

**Keywords:** acceptability, breast cancer, qualitative research, risk assessment, risk communication

## Abstract

**Background:**

Identifying women aged 30–39 years at increased risk of developing breast cancer could allow them to consider screening and preventive strategies. Research is underway to determine the feasibility of offering breast cancer risk assessment to this age group. However, it is unclear how best to deliver and communicate risk estimates to these women, in order to avoid potential harms such as undue anxiety and increase benefits such as informed decision-making.

**Objectives:**

This study aimed to investigate women’s views on, and requirements for, this proposed novel approach to risk assessment.

**Design:**

A cross-sectional qualitative design was used.

**Methods:**

Thirty-seven women aged 30–39 years with no family history or personal history of breast cancer participated in seven focus groups (n = 29) and eight individual interviews. Data were analysed thematically using a framework approach.

**Results:**

Four themes were developed. *Acceptability of risk assessment service* concerns the positive views women have towards the prospect of participating in breast cancer risk assessment. *Promoting engagement with the service* describes the difficulties women in this age group experience in relation to healthcare access, including mental load and a lack of cultural awareness, and the implications of this for service design and delivery. *Impact of receiving risk results* focuses on the anticipated impacts of receiving different risk outcomes, namely, complacency towards breast awareness behaviours following low-risk results, an absence of reassurance following average-risk results and anxiety for high-risk results. *Women’s information requirements* highlights women’s desire to be fully informed at invite including understanding why the service is needed. In addition, women wanted risk feedback to focus on plans for management.

**Conclusion:**

The idea of breast cancer risk assessment was received favourably among this age group, providing that a risk management plan and support from healthcare professionals is available. Determinants of acceptability of a new service included minimising effort required to engage with service, co-development of invitation and risk feedback materials and the importance of educational campaigning about the potential benefits of participation in risk assessment.

## Introduction

Epidemiological studies from the past decade illustrate that breast cancer incidence is increasing in pre-menopausal women worldwide.^1–3^ Younger women are more likely to have aggressive breast cancer subtypes, which lead to poorer prognosis and decreased survival despite intensive and prolonged treatment regimens.^4,5^ Breast cancer is more frequently lethal in younger women than in those diagnosed aged over 50 years (10-year survival aged <40 years at diagnosis 70% vs 87% in those >50 years).^6^ Consequently, there is a growing urgency to implement initiatives to identify younger women at higher risk so that screening and preventive strategies can be offered.^7^

In the United Kingdom, a strong family history of breast cancer is the only basis for comprehensive breast cancer risk assessment that currently allows women aged under 50 years to access screening and preventive strategies.^8^ However, at least 65% of women who develop breast cancer before the age of 50 years do not have a family history and are not currently identified as being at increased risk.^6,9^

One proposed approach to identify women at increased risk of breast cancer involves the use of personalised breast cancer risk estimates.^10^ The development of risk algorithms, such as the Gail, Tyrer–Cuzick and BOADICEA models, has made it possible to estimate an individual’s risk of breast cancer.^11–14^ These models typically include a combination of ‘classic’ risk factors including family history, hormonal and reproductive history and lifestyle. In recent years, risk prediction models have incorporated polygenic risk scores and mammographic breast density, which have improved their discriminatory accuracy.^15–18^ The merits of personalised risk assessment for breast cancer has predominantly been explored within the context of implementing risk stratification into breast cancer screening programmes, that is, tailoring of screening practices based on individual variation in risk.^19^ This approach has the potential to improve the benefit to harm ratio of screening.^20^ Recruitment is ongoing for several international trials to determine the potential effectiveness of a risk-based screening regimen in comparison to standard screening practice.^21,22^

Given the increasing burden of breast cancer in pre-menopausal women, the feasibility of offering breast cancer risk assessment to women aged 30–39 years is currently being investigated (NCT05305963). In line with the Medical Research Council (MRC) Framework for Developing and Evaluating Complex Interventions,^23^ it is important to consider acceptability to potential recipients of a new service as a major determinant of feasibility. To date, the views and attitudes of women who are too young to attend breast screening and who are not at known increased risk of breast cancer have rarely been investigated. Previous research with screening age women has demonstrated that concerns about inducing unnecessary worry and perceived ability to cope emotionally with risk feedback influence decisions to participate in breast cancer risk assessment.^24,25^ In addition, a recent multicriteria decision analysis identified service access as the most valued criteria influencing decisions to participate.^26^ However, it remains unclear how best to implement breast cancer risk assessment and communicate risk information outside of an organised screening programme to younger women.

This study aimed to investigate prospective service users’ attitudes towards a proposed new breast cancer risk assessment service to inform the delivery model adopted in the feasibility study.

Specific objectives were to:

1. Understand women’s views on, feelings about and attitudes towards introducing breast cancer risk assessment.
2. Understand women’s preferences for access to and delivery of a breast cancer risk assessment service.
3. Identify women’s information and support needs with respect to breast cancer risk assessment and risk communication.

## Methods

### Design

A cross-sectional qualitative design was used. Given the novel and hypothetical nature of the discussion topic, focus groups were chosen as the primary method of data collection. Participants were unlikely to have considered breast cancer risk assessment previously, and therefore, the debate within the group would encourage participants to reflect on and clarify their own perspectives, resulting in a depth of dialogue not often found in interviews.^27^ Focus groups also allow participants to consider their views in relation to others, which might result in opinions shifting during the course of discussion.^28^ Therefore, this interaction between participants allows insight into the degree of group consensus on the topic.^29^ One-to-one interviews were carried out when participants were unable to attend scheduled focus groups.

### Participants

Participants were recruited through responding to study advertisements outlining the inclusion criteria and topic. Inclusion criteria were chosen to ensure that the resulting sample matched the intended recipients of the planned study assessing feasibility of offering breast cancer risk assessment to women aged 30–39 years (NCT05305963). Participants met study inclusion criteria if they were (1) born female, (2) aged 30–39 years, (3) residing in Greater Manchester, (4) able to provide informed consent and (5) able to understand and communicate in the English language. Women could not take part if they had received a breast cancer diagnosis or had a first-degree female relative (mother or sister) affected by breast cancer.

### Procedure

Poster advertisements were shared on social media platforms (Facebook and Twitter) and displayed on noticeboards in public buildings (e.g. libraries and community centres). The co-founder of a community organisation made direct contact with the research team after seeing an advertisement on Twitter and facilitated the recruitment of four members of the community group who were from ethnic minority backgrounds. Prospective participants were screened against inclusion criteria via email/telephone and sent information sheets.

Topic guide development was informed by the aims of the study and a review of the literature. An initial draft was developed by the lead author, a doctoral student in health psychology with qualitative health services research experience. Feedback on this draft was obtained from public contributors and the rest of the research team who have extensive clinical and research expertise in breast cancer and screening services, medical oncology, health services research, health psychology and qualitative methods. Modifications to the content and structure of the topic guide were made in response to the feedback received. Questions focused on the acceptability of breast cancer risk assessment in relation to the process being used in the planned feasibility study. There were three proposed components of the study process, and each of these were discussed in turn: (1) a questionnaire for self-reported assessment of breast cancer risk factors, (2) a saliva sample for assessment of polygenic risk and mutations in high-risk genes and (3) a low-dose mammogram for assessment of breast density. In addition, views on methods of access, information and support needs, risk communication and anticipated barriers to engagement were captured (see Supplementary Material 1).

Focus groups and interviews were conducted between February and November 2020, either in person at a University building or in participant’s homes (interviews only) or later via telephone and Zoom conferencing due to COVID-19 restrictions. Following consent, participants gave demographic information. Participants were compensated for their time with a £20 cash payment. Focus groups and all interviews were audio-recorded and transcribed verbatim. A recording malfunction occurred during one online focus group meaning that only the first half of discussion was recorded. Field notes were taken to capture insights from the remaining discussion. Personally identifiable information was anonymised, and participants were assigned pseudonyms. Data collection was primarily conducted by the lead author (S.H.) with another member of the research team co-facilitating the focus groups (R.E.H.). Both facilitators are female researchers and have postgraduate qualitative health services research experience. Interviews were conducted by the lead author (S.H.). No one was present during the focus groups and interviews besides the participants and researchers. Data collection continued until the research team were satisfied that sufficient data had been collected to answer the research question.^30^

### Patient and public involvement

Community and patient stakeholders were involved in the design of this study. A research advisory group – consisting of Black, Asian and minority ethnic community leaders – was consulted regarding the research idea and intended recruitment procedures. Wording and terminology used in the recruitment poster and participant information sheet were revised following feedback from nine Black African women aged 30–39 years with no personal history of breast cancer. The topic guide was piloted with four White women aged 30–39 years with no personal history of breast cancer, and they advised on wording and relevance of questions, prompts and flow.

### Data analysis

Data were analysed in NVivo12 using thematic analysis with a framework approach to data management from a critical realist perspective.^31–33^ Primary data analysis was conducted by the lead author with input from L.G. and D.P.F. The analysis involved five main stages. (1) The lead author read transcripts, noting initial thoughts and impressions of the data. (2) The lead author conducted line-by-line coding on a selection of transcripts. Initial coding was deductive based on the structured questions in the topic guide to inform the design of the planned feasibility study in terms of the delivery model adopted, the information and support provided and communication of risk feedback. Inductive methods were then used to capture additional codes and context to ensure important aspects of the data were not missed. Codes were then grouped according to similarities and differences, producing categories. This resulted in a working thematic framework of codes contained within categories, which was refined in collaboration with L.G. as more transcripts were coded. (3) This thematic framework was then systematically applied to all remaining transcripts. (4) Each category was plotted onto a separate thematic matrix using the ‘framework’ feature in NVivo12, with codes presented in separate columns, and participants (cases) on separate rows. To reduce the data set into a more manageable form, data in each cell were summarised and illustrative quotations were noted (see Supplementary Materials 2–9). (5) The resulting data in the framework matrices were compared across and within cases to highlight similarities and differences. This facilitated the development of the final themes. Regular meetings were held between members of the research team (S.H., L.G. and D.P.F.) throughout analysis and interpretation to discuss explanations for findings, deviant cases and agreement on themes. The final thematic structure was agreed by the entire research team as representing participants’ views.

## Results

Sixty-one women contacted the research team expressing an interest in the study. Prospective participants were asked to confirm that they met the inclusion criteria. Twenty-four women did not respond to the research team following three contact attempts. This drop-off is likely to be attributable to the primary recruitment period coinciding with the beginning of the COVID-19 pandemic (February to March 2020). Thirty-seven women participated; seven focus groups (participant range n = 3–5) and eight interviews were conducted. Focus groups lasted between 71 and 90 min (median = 85 min) and interviews ranged from 40 to 81 min (median = 55 min). The majority of participants were White British (see Table 1). Based on participant residential postcodes, a wide range of deprivation was represented. Participants fell between Index of Multiple Deprivation deciles 1 and 10 (1 is the most deprived and 10 is the least), with a median decile of 4.^34^

**Table 1.** Sample demographics (n = 37).

Characteristic	N (%)
Age range (years)	
30–33	18 (49)
34–36	7 (19)
37–39	12 (32)
Ethnicity	
White British	29 (78)
Black African	4 (10)
Indian	1 (3)
White (other)	1 (3)
Mixed (White/Arab)	1 (3)
Mixed (English Caribbean)	1 (3)
Deprivation decile^a^	
High (1–3)	15 (41)
Medium (4–7)	18 (49)
Low (8–10)	4 (10)

a Assessed by participant residential postcode using the Index of Multiple Deprivation 2019, a measure of relative deprivation for small areas in England.^34^

Data are presented as four overarching themes with seven sub-themes (Table 2). Quotes are presented with a pseudonym followed by interview (I) or focus group (FG) number.

**Table 2.** Thematic structure.

Theme	Subtheme
Acceptability of risk assessment service	–
Promoting engagement with the service	Fitting with high mental load
	‘It’s community effort’
Impact of receiving risk results	Avoiding complacency towards breast awareness following a low-risk result Questioning the impact of an average-risk result Mitigating anxiety following a high-risk result
Women’s information requirements	Enabling informed decision-making at invite
	Recommendations for risk communication

### Theme 1: acceptability of risk assessment service

The majority of women stated they would welcome the opportunity to find out their breast cancer risk. However, some women expressed reservations, citing concerns about the potential for acute emotional distress while awaiting results and significant impacts on mental health thereafter:

my first thought is, do you know what, I don’t think I want to know because then that would just give me anxiety. . . I think the anxiety would probably kill me before the cancer does. (Rebecca, FG6)

Participants contextualised the risk assessment process by reflecting on previous encounters with healthcare, for example, cervical screening, pregnancy and childbirth. Consequently, participants were accepting of the questionnaire, saliva sample and mammogram as they were not regarded as invasive procedures. The content of the risk factors questionnaire was considered reasonable due to the factual, medical nature of questions. However, reliance on self-report was flagged as a potential concern in relation to how incorrect recall of information may affect accuracy. Providing a saliva sample for genetic testing was considered commonplace and therefore acceptable due to availability of direct-to-consumer genetic testing from companies such as 23andMe. The most frequently reported concern about the risk assessment process was anticipated discomfort during the mammogram. This was influenced by older relatives’ anecdotal experiences of mammograms being uncomfortable and painful.

The main benefit of participating in breast cancer risk assessment was perceived to be the ability to plan and prepare for the future in terms of accessing breast cancer risk management strategies to maintain good health:

if you know what you’re facing you can deal with it, can’t you? You can say, ‘Oh well, that’s fine, we’ll do this, this and this, and we can have a plan of action’. (Grace, I4)

Participants were generally aware of the importance of early detection of cancer for increased chances of survival and as such, viewed the service favourably as an opportunity to detect breast cancer earlier and thus reduce breast cancer mortality. Willingness to undergo risk assessment was contingent on there being options to reduce or manage the risk, preferably with the support of healthcare professionals. There was limited awareness of preventive measures among participants. However, when prompted, many women were uncomfortable with the prospect of taking medication for reduction of risk in the absence of disease:

the most common thing you take medication for is if there’s something wrong with you right now. So it would almost make you feel like there was I guess. (Florence, FG5)

In contrast, one woman stated she would find medication a reassuring and preferable option to lifestyle advice, owing to the strong motivation required to change health-related behaviours.

### Theme 2: promoting engagement with the service

This theme describes the difficulties women experience accessing existing healthcare services and the implications of this for service design and delivery.

#### Subtheme 2a: fitting with high mental load

Women perceived the proposed timing of invitation to the breast cancer risk assessment service to coincide with a defining decade in terms of managing the often competing demands of professional and family life:

it’s not unusual for people at my age to have one, two or three children of a certain age. So your time’s already spread a bit thin, it would be spread even thinner especially if you’ve got multiple children, a job, a house, things like that. (Ashleigh, I3)

As a result, women reported being time-poor and experiencing difficulties accessing healthcare services at a time that suited them. In addition to physical access requirements, women described a high mental load in the form of invisible labour involved in managing a household and family, which often results in putting the needs of others before their own:

As a group of women in their 30s, often it can be other people’s health, other things going on and we’re often at the bottom of the pile. (Gemma, FG4)

Therefore, women desired a ‘one-stop shop’ delivery model whereby all three components of breast cancer risk assessment would be completed at one appointment:

Get it all done in one go, yeah, and then you don’t have to use brain space for it twice. (Laura, I1)

Participants stated they would be more likely to engage with the service if it was available in their local area. Poor public transport routes to hospitals and the resources needed to travel were identified as potential barriers to engagement. To overcome these issues, some women expressed a preference for a mobile service in community settings such as workplace and supermarket car parks.

Availability of appointments outside of normal working hours was considered an essential requirement of the service. Furthermore, the ability to book a convenient appointment online or via phone was desired to reduce the cognitive burden of re-arranging a pre-specified appointment:

if they can book it themselves online or over phone or whatever option is available to them something that they can select the date and the time [. . .] that would be really helpful if they were able to do it themselves rather than getting told you need to be here on this date and this time. (Danielle, I8)

#### Subtheme 2b: ‘it’s community effort’

Involvement of community leaders in service delivery was considered vital. Participants described how community leaders often share the culture and language of the community they support and this shared background helps to build rapport. In addition, community leaders were perceived to possess cultural awareness of beliefs and perceptions women may have when considering engagement with healthcare services. For example, one participant described how young women in her community worry about attending cervical cancer screening for fear of bringing dishonour to their families because of the intimate nature of the test. Participants explained how services could work with community leaders to tailor health messaging in a more meaningful and sensitive way, which women felt would increase the likelihood of engagement, particularly for women from ethnic minority backgrounds:

if you’ve got community groups or community leaders involved they know how to talk, how to communicate the information to the people of their community [. . .] they know there’s this misconception or there’s fear [. . .] and they are able to address those sensitivities that the healthcare industry might not actually be aware of. (Tara, FG6)

### Theme 3: impact of risk results

This theme captures participants’ perceptions of anticipated impacts following receipt of low, average and high-risk results. The labelling of risk results reflects the terminology the women used when asked for their thoughts about the use of risk categories.

#### Subtheme 3a: avoiding complacency towards breast awareness following a low-risk result

Participants expressed reservations about provision of a low-risk result. It was thought that women labelled as low-risk may no longer perceive breast cancer as a pertinent concern, which could result in complacency towards breast awareness behaviours. One woman perceived complacency as an inevitable consequence of labelling a woman as low-risk and as such felt the word ‘low’ should not be used:

scrap the language so you don’t use the word low risk because that would lead to complacency in behaviours. (Gemma, FG4)

Examples of possible complacency reported by women included losing vigilance around breast awareness and reluctance to seek help for breast symptoms. To avoid these potential negative impacts, women thought it would be necessary to make sure those labelled as low-risk understood that the result did not guarantee they would never develop breast cancer:

people need to be made aware that just because it’s low doesn’t mean it’s not impossible. (Natasha, FG4)

#### Subtheme 3b: questioning the impact of an average-risk result

Participants discussed the possibility of a risk category between low and high indicating average risk for the population. Some women questioned the value of receiving such a result with one woman describing it as the ‘worst category to fall into’ (Tiffany, I6). For these women, it was unclear what management strategies would result from this level of risk:

So if you are gonna tell somebody they’re of average risk is there still options to prevent it [. . .] or is average not good enough to prevent. (Brittany, FG7)

Furthermore, women felt they would not have gained any new knowledge from participating in the risk assessment service if they received an average-risk result:

I feel like I’d be in the same boat now, you, kind of, don’t know either way do you? You don’t know, there’s a chance you could but there’s a chance you might not whereas that would be our position now. (Debbie, FG5)

Consequently, an average-risk result was considered to hold no meaning and in turn, women were not reassured by this result. One woman suggested it might make her feel panicked:

if the average risk if it was just like oh well there’s not much you can do just keep checking your breasts I might be a bit I don’t know. I don’t know if I’d get panicked about that or not. (Brittany, FG7)

#### Subtheme 3c: mitigating anxiety following a high-risk result

In anticipation of significant anxiety following a high-risk result, participants welcomed the prospect of attending a clinic appointment to discuss their risk result further. However, feelings of vulnerability and apprehension were expected in anticipation of attending this appointment. Women thought attending a hospital, particularly one specialising in oncology, would imply current illness, which would lie in tension with the intended purpose of the appointment being to reduce the risk of developing breast cancer in the future. Furthermore, women felt the name of the clinic needed careful consideration as inclusion of the word ‘risk’ implied threat. These aspects of the appointment are important to consider, as they may increase women’s perceived threat to a level that results in disengagement with risk management:

can you imagine being invited back to a breast centre . . . you’d be thinking ‘right I’m a cancer patient’. (Debbie, FG5)

During the clinic appointment, women wanted to discuss the implications of their risk result for their children’s lives, particularly daughters. One woman thought a high-risk result could be ‘life-changing information’ as it could potentially influence reproductive decision-making:

if you found out you were super high-risk and you were a very logical person, you might just go, okay, well, I’m not going to do that [have children] because if there’s a chance that within the next ten years my life’s going to be on the line, I don’t want to bring kids into the world only to then leave them. (Kathryn, I7)

In addition to a clinic appointment, a few women desired a peer support group. For these women, an interaction with a healthcare professional was considered inadequate in fulfilling emotional support needs. Peer support was valued as an opportunity for women with shared experience to connect and learn from each other.

### Theme 4: women’s information requirements

This theme describes the information participants expected to be included in invitation and risk feedback material.

#### Subtheme 4a: enabling informed decision-making at invite

Women expected to be informed about evidence supporting the introduction of the service and the benefits of participation. This need was rooted in women’s beliefs and assumptions of breast cancer and screening. Participants assumed that screening is offered to women when breast cancer poses the greatest risk. Consequently, women perceived breast cancer as a disease predominantly affecting women older than 50 years:

I think the fact that they don’t even offer mammograms to anyone under 50, kind of, it automatically makes you think that therefore it’s not a risk to you, you know, it’s like oh the authorities have decided that that’s the age that you’re likely to get it therefore if you’re younger than that like don’t worry about it almost. (Brooke, FG3)

The ‘helping you decide’ leaflet accompanying cervical cancer screening invitations was considered a good exemplar of information giving. Some women desired a simple diagrammatic representation of the different pathways possible from engaging with the service.

Participants with professional experience of designing and producing healthcare information thought that information should be made available in different languages and formats such as easy read and braille to increase accessibility and equity of access. One woman from an ethnic minority background felt that more ethnically diverse images should be used in information materials to convey the importance of engaging with the service irrespective of cultural or racial background:

maybe putting more information with people that look like the communities that you’re trying to reach [. . .] it becomes more personal to them like ‘oh wow, if someone who looks like me has been affected by it, maybe I need to take this seriously’. (Rebecca, FG6)

Participants wanted access to a video resource showing a woman having the mammogram procedure. Perceived benefits of this approach included women feeling more reassured and prepared for what to expect and making the procedure easier to imagine:

usually if it’s to do with the actual procedure I prefer looking at it and sort of seeing it visually happen so I can imagine myself going through that. Whereas if you’re just telling me over the phone, ‘Oh, well, you just go into a room, then you get your bra off and stand at a machine’, I can visualise it but I don’t know how I would react in that situation. (Ashleigh, I3)

Women expected to be informed about timescales of activity within the service. For example, the duration between completing the risk assessment process and receiving results. A few women said they would feel more comfortable attending the clinic appointment if it was made clear in the invitation material that the radiographer performing the mammogram was a woman:

women that come from maybe religiously conservative backgrounds, they might want to be absolutely reassured that they’re going to be seen like in a women only environment. (Tiffany, I6)

Women wanted to be informed about the intended scope of genetic testing. This included information on how DNA would be stored and shared as a means of reassurance that personal information was safe.

Participants were familiar with the use of mammograms in breast cancer screening to detect breast cancers. Therefore, participants presumed the risk assessment mammogram would have diagnostic capability and found it difficult to understand why the presence or absence of a breast cancer could not be confirmed at the same time as assessment of breast density. For this reason, explicit communication of the purpose of the mammogram in the invitation materials was considered essential to avoid any misunderstandings:

I think you would assume that a mammogram would pick up on cancer if it was there [. . .] I guess that’s a really important message to get out there if you do this isn’t it, this isn’t a replacement for a cancer screening. (Laura, I1)

#### Subtheme 4b: recommendations for risk communication

Women described not wanting to be caught off guard when receiving their risk result. For example, receiving advanced notice of a phone call. This can be seen to function as a means of retaining some control in an uncontrollable situation:

Do I want to be ambushed on the phone with someone telling me that I should go and discuss my breast cancer risk? (Roisin, FG1)

For high-risk results, women preferred a telephone call or face-to-face consultation with a healthcare professional as this would allow the opportunity to ask questions immediately, mitigating the anticipated emotional distress to receiving a high-risk result. It was acknowledged by some women that being in this heightened emotional state may interfere with cognitive processing of information and as such, written information to refer back to was desired. In contrast, receiving a low-risk result via letter or text message was considered acceptable.

Irrespective of risk result, all participants expected to be given the option of accessing a healthcare professional with appropriate expertise. General practitioners were perceived as lacking the required expertise needed.

Overall, there was a lack of consensus regarding the optimal method of displaying risk information. Women thought multiple formats of risk presentation should be offered to accommodate different learning styles and low levels of literacy and numeracy.

Women assumed that each risk category would span a broad range of percentages. Consequently, women perceived knowing where they were in the category as a more informative result. In contrast, one woman felt that provision of the risk category was sufficient and easier to process from an emotional point of view. Providing comparative risk information in the form of the national average was considered beneficial to contextualise the severity of the risk result:

It [national average] kind of creates a bit of understanding doesn’t it in your mind [. . .] what that actually means in terms of your increased risk. (Gemma, FG4)

Participants expressed reservations about using colour to differentiate between risk levels. In particular, strong emotional reactions were observed in response to the suggestion of using red to denote high risk:

Jasmine: If I was a high risk and I saw red, I’d be like, ‘Oh, I’m going to die now’. [Laughter] And it would probably be a bit off-putting.

Carrie: Yeah, ‘High alert. Death round the corner’. [Laughs].

Nancy: Yeah, ‘Your boobs are going to fall off’. (FG2)

In terms of risk feedback, information about the factors contributing to risk was perceived as interesting but generally unhelpful due to the perception of many breast cancer risk factors as non-modifiable. Instead, information about what would happen next in terms of proactive risk management was of utmost importance to all participants:

I would want it to be focussed on what I do next and not on what the result is. Because if the result’s scary, then I just want to know and then I want to move on and know what to do next. (Tiffany, I6)

Participants expected risk feedback to contain information on lifestyle recommendations because receiving risk was considered an opportune time for women to review and modify unhealthy lifestyle behaviours such as smoking and excessive alcohol consumption. Furthermore, guidance on how to perform breast checks and recommended frequency of doing so was desired.

## Discussion

To our knowledge, this is the first study to investigate views of young women, not known to be at increased risk of breast cancer, regarding a proposed novel approach to risk assessment outside of an organised breast screening programme. Overall, women responded positively to the prospect of a breast cancer risk assessment service. However, the findings illustrate certain conditions of acceptability that will be key considerations in implementation. Participants desired a ‘one-stop shop’ delivery model whereby all components of a breast cancer risk assessment would be completed at a single appointment. Various concerns were apparent when anticipating receiving all risk results, not just results indicating high risk. Women expressed low perceived susceptibility to developing breast cancer and reported limited awareness of breast cancer as a potentially preventable disease. Consequently, women wanted to be fully informed ahead of engaging with breast cancer risk assessment. Verbal communication methods (telephone and in person) were preferred for high-risk results to mitigate any induced emotional distress. In comparison, a letter or text message was deemed acceptable for low risk. In terms of risk presentation, women advocated the use of multiple formats that combine quantitative and qualitative features. Overall, presentation of risk was considered less important than implications of the result for risk management. Irrespective of risk result, women expected to be given the option of support from a healthcare professional with appropriate expertise.

### Strengths and limitations

The sample matched the intended recipients of the feasibility study. Adopting this approach allowed the needs of the communities who are eligible to participate to be identified, increasing the likelihood of successful implementation in the feasibility study and subsequent wider implementation. In addition, we were interested in including the voices of Black British women as they are at greater risk of developing breast cancers with less favourable characteristics, at younger ages, resulting in reduced survival.^35,36^ The inclusion of Black women is considered a strength of this study as their voices are typically under-represented in cancer prevention and early detection research.^37^ However, we recommend that future research focuses on examining Black women’s views in greater detail.

Limitations include that many participants were motivated to participate in the study having witnessed the negative impacts of a peer or second-degree relative’s breast cancer diagnosis. These experiences might have resulted in more positive views of breast cancer risk assessment, leading to overestimations of acceptability. However, participants were given the opportunity to express positive and negative views, and particular attention was given to dissenting voices during data analysis.

Second, women were responding to the idea of a hypothetical breast cancer risk assessment service meaning their responses reflect anticipated thoughts and feelings. Nevertheless, although women may respond differently if the service is implemented in clinical practice, the approach taken is the most appropriate, given that the present research is designed to inform a new service that does not currently exist.

Finally, we did not assess educational attainment or health literacy of the participants, which is likely to have influenced the findings particularly in relation to information requirements. Women with low health literacy may have different views and preferences with respect to the information required, and this should be taken into account when developing invitation and risk feedback materials.

### Relevance to existing literature

The ease of accessing a breast cancer risk assessment service was identified as a critical determinant of engagement, leading to women desiring a ‘one-stop shop’ delivery model. Women reported being unable to attend healthcare appointments due to a lack of time and service availability constraints. This finding is in line with previous research exploring non-attendance at other healthcare services such as cervical cancer screening.^38,39^ Women also described a high mental load, defined as the cognitive and emotional labour involved in managing a household and family life.^40^ In addition, women recommended involving community leaders in service delivery to increase engagement of women from ethnic backgrounds which has been found to be an effective approach among ethnic minority groups for improving uptake to cancer screening programmes.^41^

Consistent with previous research,^42,43^ women expressed concern about provision of a low-risk result for fear it could result in complacency towards breast awareness behaviours such as performing breast checks. One study found that receipt of risk information did not significantly change low-risk women’s intentions to attend subsequent mammograms.^44^ However, it remains unknown how receipt of a low-risk result at a younger age may affect breast awareness behaviours and subsequent engagement with breast screening.

This study offers novel insight into anticipated appraisal of average-risk feedback, which has received little attention in previous research. The findings suggest women would not feel reassured by an average-risk result. The purported benefits of receiving low- and high-risk results have been reported by women in previous studies.^45,46^ However, women in this study were unable to identify a tangible benefit of receiving an average-risk result.

### Implications and future research directions

Given the high mental load women report experiencing which negatively affects healthcare access, a ‘one-stop shop’ delivery model that minimises the physical and cognitive energy required to engage with breast cancer risk assessment would be an attractive approach to implementation.

The findings suggest that women may not understand the value in engaging with breast cancer risk assessment because of their beliefs and assumptions of breast cancer and screening. Therefore, it is imperative that information is provided in the invitation materials to assure women of the need for the service. Furthermore, a mass media education campaign should be delivered prior to and during implementation to help women understand why it is important for them to consider engaging with the service. Invitation and risk feedback materials need to be co-developed and piloted with women to ensure information requirements identified in this study are addressed and informed choices can be made about participation. Future qualitative research should examine the views and needs of diverse communities in terms of protected characteristics, socioeconomic variability, health literacy and other geographical areas to inform localised approaches to implementation. This research would benefit from adopting an intersectional approach to reduce health disparities among marginalised populations in subsequent implementation research.^47^

This study also highlighted the possibility of harmful effects from communicating low-risk estimates. Implementation studies should consider assessing the long-term impact of communicating risk estimates on breast awareness behaviours such as performing breast checks. In addition, appraisals of average-risk feedback warrant further attention to determine the purpose and utility of providing such information if not perceived as reassuring.

## Conclusion

This research has demonstrated that a breast cancer risk assessment service is acceptable for women aged 30–39 years providing that a risk management plan and support from healthcare professionals with appropriate expertise is available. Minimising the effort required to engage with the service has been identified as a key determinant of acceptability. There is a need for co-development of invitation and risk feedback materials and educational campaigning about the potential benefits of risk assessment to facilitate informed decision-making about participation.

## Supplemental Material

sj-docx-1-whe-10.1177_17455057231160348 – Supplemental material for Optimising the delivery of breast cancer risk assessment for women aged 30–39 years: A qualitative study of women’s views Click here for additional data file. Supplemental material, sj-docx-1-whe-10.1177_17455057231160348 for Optimising the delivery of breast cancer risk assessment for women aged 30–39 years: A qualitative study of women’s views by Sarah Hindmarch, Louise Gorman, Rhiannon E Hawkes, Sacha J Howell and David P French in Women’s Health

## Declarations

**Ethics approval and consent to participate:** Ethical approval was received from the University Research Ethics Committee, University of Manchester (reference 2019-7900-12761) and Health Research Authority (HRA), and the study was performed in accordance with the Declaration of Helsinki, Good Clinical Practice principles and relevant regulations. All participants provided informed written or verbal consent prior to taking part in the study. Verbal consent was obtained for participants of online focus groups and telephone interviews using a consent script owing to logistical difficulties obtaining written consent during the COVID-19 pandemic. Verbal consent was audio-recorded separately to the focus group or interview.

**Consent for publication:** All participants provided consent for publication and gave approval for quotations from their transcripts to be published.

**Availability of data and materials:** The author(s) confirm that the data supporting the findings of this study are available within the article and its supplementary materials.
