# Supplementary material for: Optimising the delivery of breast cancer risk assessment for women aged 30–39 years: A qualitative study of women’s views
Source: Womens Health (Lond). 2023 Mar 31;19:17455057231160348. doi: 10.1177/17455057231160348 (PMC10071144; doi:10.1177/17455057231160348)
Supplement: sj-docx-1-whe-10.1177_17455057231160348 – Supplemental material for Optimising the delivery of breast cancer risk assessment for women aged 30–39 years: A qualitative study of women’s views [file sj-docx-1-whe-10.1177_17455057231160348.docx]

**Supplementary material information**

File name: Supplementary material 1
Legend: Focus group and interview topic guide
Description: A list of the questions asked during focus groups and interviews

File name: Supplementary material 2
Legend: Matrix 1: Acceptability of breast cancer risk assessment
Description: Thematic matrix for acceptability data, with codes presented in separate columns, and participants (cases) on separate rows

File name: Supplementary material 3
Legend: Matrix 2: Methods of access to breast cancer risk assessment
Description: Thematic matrix for methods of access data, with codes presented in separate columns, and participants (cases) on separate rows.

File name: Supplementary material 4
Legend: Matrix 3: Information needs ahead of breast cancer risk assessment
Description: Thematic matrix for information needs data, with codes presented in separate columns, and participants (cases) on separate rows

File name: Supplementary material 5
Legend: Matrix 4: Presentation and content of risk feedback
Description: Thematic matrix for risk feedback data, with codes presented in separate columns, and participants (cases) on separate rows

File name: Supplementary material 6
Legend: Matrix 5: Preferences for receipt of risk information
Description: Thematic matrix for receipt of risk information data, with codes presented in separate columns, and participants (cases) on separate rows

File name: Supplementary material 7
Legend: Matrix 6: Preferences for accessing support
Description: Thematic matrix for preferences for accessing support data, with codes presented in separate columns, and participants (cases) on separate rows

File name: Supplementary material 8
Legend: Matrix 7: Anticipated barriers to breast cancer risk assessment
Description: Thematic matrix for barriers data, with codes presented in separate columns, and participants (cases) on separate rows

File name: Supplementary material 9
Legend: Matrix 8: Risk perceptions
Description: Thematic matrix for risk perceptions data, with codes presented in separate columns, and participants (cases) on separate rows
